# Intensity-modulated radiotherapy for prostate cancer with seminal vesicle involvement (T3b): A multicentric retrospective analysis

**DOI:** 10.1371/journal.pone.0210514

**Published:** 2019-01-25

**Authors:** Flora Goupy, Stéphane Supiot, David Pasquier, Igor Latorzeff, Ulrike Schick, Erik Monpetit, Geoffrey Martinage, Chloé Hervé, Bernadette Le Proust, Joel Castelli, Renaud de Crevoisier

**Affiliations:** 1 Radiation Department, CLCC Eugène Marquis,Rennes, France; 2 Radiation Department, CLCC René Gauducheau, Nantes, France; 3 Radiation Department, CLCC Oscar Lambret, Lille, France; 4 Radiation Department, Clinique Pasteur, Toulouse, France; 5 Radiation Department, University Hospital Cavale Blanche, Brest, France; 6 Radiation Department, Clinique Saint-Yves, Vannes, France; 7 Medical Imaging Department, CLCC Eugène Marquis, Rennes, France; 8 University Rennes 1, LTSI (Laboratoire Traitement du Signal et de l'Image), Inserm U1099, Rennes, France; North Shore Long Island Jewish Health System, UNITED STATES

## Abstract

**Objectives:**

No study has reported clinical results of external-beam radiotherapy specifically for T3b prostate cancer. The possibility of escalating the dose to the involved seminal vesicles (ISV) while respecting the dose constraints in the organs at risk is thus so far not clearly demonstrated. The objective of the study was to analyze the dose distribution and the clinical outcome in a large series of patients who received IMRT for T3b prostate cancer.

**Materials and methods:**

This retrospective analysis included all patients who received IMRT and androgen deprivation therapy for T3b prostate cancer, between 2008 and 2017, in six French institutions, with available MRI images and dosimetric data.

**Results:**

A total of 276 T3b patients were included. The median follow-up was 26 months. The median (range) prescribed doses (Gy) to the prostate and to the ISV were 77 (70–80) and 76 (46–80), respectively. The dose constraint recommendations were exceeded in less than 12% of patients for the rectum and the bladder. The 5-year risks of biochemical and clinical recurrences and cancer-specific death were 24.8%, 21.7%, and 10.3%, respectively. The 5-year risks of local, pelvic lymph node, and metastatic recurrences were 6.4%, 11.3%, and 15%, respectively. The number of involved lymph nodes (≤ 2 or ≥ 3) on MRI was the only significant prognostic factor in clinical recurrence (HR 9.86) and death (HR 2.78). Grade ≥ 2 acute and 5-year late toxicity rates were 13.2% and 12% for digestive toxicity, and 34% and 31.5% for urinary toxicity, respectively. The dose to the pelvic lymph node and the age were predictive of late digestive toxicity.

**Conclusion:**

IMRT for T3b prostate cancer allows delivery of a curative dose in the ISV, with a moderate digestive toxicity but a higher urinary toxicity. Lymph node involvement increases the risk of recurrence and death.

## Introduction

T3b prostate cancer (PCa) stage is defined as cancer invasion of one or both seminal vesicles (SV) [1]. Even if the T staging of the D’Amico risk group classification [2] is based on digital rectal examination (DRE), MRI is recognized as the most accurate modality to stage PCa. Indeed, if MRI has a low sensitivity to detect the involved SV (ISV) (around 60%), its specificity is particularly high (around 95%) [3]. The prediction of ISV, confirmed by surgery, is thus statistically increased by adding MRI to clinically based models such as Partin tables or CAPRA score [4,5]. On MRI, the incidence of T3b stage is reported to be around 10% [6,7]. After radical prostatectomy, pathological ISV ranged from 5% to 18%, depending on patient selection [8–15].

The standard of care for locally advanced PCa is radiotherapy (RT) combined with 2 to 3 years of androgen deprivation therapy (ADT) [16,17]. The increasing number of T3b PCa cases, due to the recent generalization of multiparametric MRI, raises, however, the issues of both the radiation technique and its clinical results for these specific diseases. Indeed, delivering a high curative dose in the ISV [18] is particularly challenging for respecting the dose constraints in the rectum and bladder [19–23]. In this context, IMRT is a particularly relevant tool to optimize the dose distribution [24,25]. To our knowledge, no study has reported clinical results of external-beam RT and, in particular, IMRT specifically for T3b cancer. The possibility of escalating the dose to the ISV while respecting the dose constraints in the organs at risk (OAR) is thus so far not clearly demonstrated.

The objectives of this study were, in cases of IMRT for T3b PCa, to analyze retrospectively the planned dose to the ISV and the OARs and the patient outcome in a large series of patients.

## Patients and methods

### Inclusion criteria, initial staging, treatment, and follow-up

Our retrospective analysis included all patients with T3b N0-1 M0 prostate adenocarcinoma who received IMRT at a curative dose (≥ 70 Gy) in combination with ADT, in six French institutions (Rennes, Nantes, Lille, Toulouse, Brest, and Vannes) between 2008 and 2017, with available MRI images and dosimetric and clinical data. Only patients with ISV on pre-therapeutic MRI were included. All patients had prostate biopsy showing adenocarcinoma. SV biopsy was performed in a subset of patients only, depending on the urologists. The initial staging comprised digital rectal examination (DRE), PSA measurements, Gleason score, pelvic MRI, and Tc99m bone scintigraphy.

The multiparametric MRI included T1- and T2-weighted (T1- and T2-WI), diffusion-weighted imaging (DWI), and dynamic, contrast-enhanced imaging with a 1.5 Tesla magnet without endorectal coil. ISV was defined on T2–WI images as SV disruption or loss of the normal architecture, focal or diffuse areas of low signal intensity, low signal intensity causing a mass effect, or evident tumor at the prostate base extending to the SV. ISV was defined on DWI images as low focal signal lesions, relative to benign prostate tissues on apparent diffusion coefficient maps, within the SV. ISV was defined on dynamic T1–WI as symmetric or irregular SV wall enhancement ISV [26]. The length of ISV was defined on MRI, considering the three thirds of SV in the cranio-spinal axis. Finally, the lymph node involvement (N1) was defined as a pelvic node ≥ 8 mm in the smallest diameter [27]. Approximately 50% of MRI images were subjected to a second independent reading by an expert radiologist.

Patients underwent simulation and treatment in the supine position. Intravenous iodine contrast was required. At the time of simulation, there were no recommendations concerning bowel or bladder filling. During treatment, patients had to keep a full bladder. The target volume and organs at risk were delineated on CT slices, whose spacing was 2 or 3 mm. The target delineation and dose distribution were performed according to the French Study Group on Urogenital Tumors (GETUG) guidelines [21]. The clinical target volume (CTV) included systematically the whole prostate and the non-involved SV and ISV. The SV were therefore divided in three equal thirds, according to the cranio-spinal axis. The prophylactic irradiation of the pelvic lymph nodes, performed in 90% of cases, depended on the choice of the radiation oncologist. The pelvic lymph nodes were systematically irradiated in case of involvement of the pelvic lymph nodes at MRI. The pelvic lymph node CTV included the obturator, presacral, external, internal and common iliac lymph nodes. The planning target volume (PTV) margins were defined as 5 to 10 mm around the prostate and SV, except in the posterior direction, where the margin was limited to 5 mm, and 7 mm around the pelvic lymph node. The total dose delivered to the prostate (PTV) ranged from 70 to 80 Gy at a standard fractionation of 2 Gy/fr during 7 to 8 weeks, in 87% of patients. In one center (13% of the patients), the prostate total dose was 74.8 Gy, at a fractionation of 2.2 Gy/fr in 6.8 weeks using a simultaneous integrated boost (SIB) technique. The total dose to the non-involved SV and ISV had range from 46 to 80 Gy, depending on the choice of the radiation oncologist. The prophylactic total dose to the pelvis ranged from 46 to 50 Gy at 2 Gy/fr in 87% of patients. This total dose was 54.4 Gy at 1.6 Gy/fr in 13% of patients, using the SIB technique. A boost could be delivered to the involved lymph nodes (at MRI) up to 74 Gy, depending also on the radiation oncologist. The rectal wall was generated with a thickness of 5 mm from the external, manually delineated rectal contour. The rectal length was defined as 1 cm from both sides of the prostate and SV PTV. The bladder wall was generated with a thickness of 7 mm from the external, manually delineated bladder contour. The dose volume histogram had to respect the GETUG recommendations [21] for the rectum (V60 Gy < 50%, V72 < 25%, maximum dose < 76 Gy), for the bladder (V60 Gy < 50%, V70 < 50%, maximum dose < 80 Gy), and for the femoral heads (V55 < 5%). IMRT could use 6 to 23 MV X-ray beams.

The follow-up consisted of clinical assessments and PSA measurements repeated every 6 months. Imaging (pelvic MRI, thoraco-abdominal computed tomography and bone scintigraphy) was performed in cases of biochemical progression or suspicion of clinical recurrence.

### Endpoints and statistical analysis

The dosimetric charts were analyzed to report the doses delivered toward the PTV and to the CTV of the prostate, uninvolved SV, and ISV (considering each third of the SV), and uninvolved and involved pelvic lymph nodes. The doses to the rectum and bladder were reported according to the reference points of the GETUG [21], RTOG [22], and QUANTEC [20]. Pearson correlation coefficients (r) were calculated to search for correlation between the doses to the OARs and target volume parameters (volume, length of the ISV, or doses). Significant correlations with r > 0.5 only were reported.

The follow-up was defined as the time between the first day of RT and the latest news. Follow-up was calculated using reverse Kaplan-Meier estimation. Biochemical recurrence was defined as a PSA level equal to or greater than the PSA nadir plus 2 ng/mL or the initiation of salvage androgen deprivation therapy (ADT) [28]. Clinical recurrence was defined by local recurrence, pelvic lymph node recurrence, or metastasis. Disease-free survival (DFS) was defined as the time between the first day of RT and one of the first following events, when occurring: biochemical recurrence, clinical recurrence, or death.

Acute and late digestive and urinary toxicities were retrospectively scored in accordance with the Common Terminology Criteria for Adverse Events (CTCAE), version 4.03. The toxicities were acute when they occurred within 90 days after the start of RT and late otherwise.

Kaplan-Meier analysis was used to calculate the risk of recurrences, deaths, and late urinary or digestive toxicity (grade ≥ 2). A logistic regression test was used to identify predictors of acute toxicity in univariate and multivariate analyses. Univariate and multivariate Cox regression analyses were performed to identify predictors of recurrences, deaths, and late toxicity. Significant parameters (defined as p value ≤ 0.05) identified in univariate analyses were tested in multivariate analyses. The predictive performance of the models was estimated by the area under the receiver operating characteristic (ROC) curve (AUC) for the logistic regression model and by the C-index for the Cox model. The following parameters were tested for recurrences and survivals: treatment center; age; T stage at DRE; PSA; number of D’Amico risk factors; MRI parameters such as extracapsular extension, bilateral prostate, or SV involvements; proximal ISV only; whole ISV; number of segment ISV; pelvic lymph node involvement; number of involved lymph nodes (on MRI); pathological parameters such as Gleason score, number of positive biopsies in the prostate, positive biopsies in SV when performed; dosimetric parameters such as the doses prescribed to the target volumes; and ADT duration. The following parameters were tested for toxicity: treatment center; age; arterial hypertension; cardiovascular disease; diabetes; anticoagulant treatment; history of pelvic surgery; T stage at DRE; MRI parameters such as bilateral SV involvement, proximal ISV only; whole ISV; number of segment ISV, and pelvic lymph node involvement; dosimetric parameters such as the doses prescribed to the target volumes and to the OARs; and ADT duration. Analyses were performed in the whole series and considering only the patients without pathological pelvic lymph nodes at MRI.

Statistical analyses were performed using the Statistical Package for the Social Sciences (SPSS) version 20.0 (IBM Corp., Armonk, NY, USA), and R version 3.4.3 (GNU Project, Lucent Technologies).

The research has been approved by the institutional review board of the Eugene Marquis Cancer Center.

## Results

### Patients, tumor and treatment characteristics

Our screening found 12% of T3b patients among all the irradiated patients for localized PCa at a curative intent (≥ 70 Gy). Among a total of 309 patients identified as having received EBRT at a curative intent for T3b PCa, 30 patients were excluded because of having been treated by three-dimensional conformal radiotherapy (3D-CRT) technique and 3 patients because the dosimetric chart was not available. A total of 276 patients were finally included. Patients and tumor characteristics are detailed in Table 1. Details are provided regarding MRI tumor characteristics.

**Table 1:** Patient and tumor characteristics.

**PATIENTS CHARACTERISTICS**	
Age (years) *	69 (42–86)
Comorbidities (%)	
Arterial hypertension	41
Cardiovascular disease	23
Diabetes	13
Anticoagulant treatment	29
History of pelvic surgery	31
**D’AMICO TUMOR CHARACTERISTICS**	
PSA (ng/mL) *	27 (2–217)
T Stage at DRE (%)	
T1	11
T2	35
T3	54
Gleason score (%)	
≤ 6	7
7 (3+4)	27
7 (4+3)	24
8	24
9–10	18
Number of D’Amico high risk factor (%)	
One (T3 only)	21
Two	65
Three	14
**BIOPSY CHARACTERISTICS**	
Number of biopsies in the prostate *	12 (2–24)
Number of positive biopsies in the prostate *	8 (1–21)
Patients with SV biopsies (%)	8
Patients with positive SV biopsies when SV biopsy was performed (%)	86
**MRI TUMOR CHARACTERISTICS (%)**	
Extracapsular extension	73
Bilateral prostate involvement	68
Bilateral involved SV	41
Length of involved SV	
Proximal 1/3 only	64
Proximal 2/3 only	20
Entire SV	16
**LYMPH NODE INVOLVEMENT°**	
Number of patients (%)	26
Number of involved node per patient *	3 (1–16)
Bilateral involvement (%)	48
Localization (%)	
Pararectal	14
Presacral	10
Obturator	29
Intern iliac	45
Extern iliac	43
Common iliac	20

*Mean value (range). DRE: digital rectal examination; SV: seminal vesicle;° defined as pelvic node ≥ 8 mm in the smallest diameter on MRI.

The median (range) prescribed doses (Gy) were 77 (70–80) for the prostate, 50 (46–80) for the uninvolved SV (on MRI), 76 (46–80) for the ISV, 50 (46–55) for the uninvolved pelvic lymph nodes, and 52 (46–74) for the involved lymph nodes (on MRI). The prescribed doses to the uninvolved SV and ISV were equal to or greater than 70 Gy for 22% and 64% of patients, respectively. The pelvic lymph nodes without lymph node involvement were treated at a prophylactic dose in 90% of cases. The mean radiotherapy duration was 56 days (range: 14–77).

The IMRT technique was based on the use of a five-field beam in 30% of patients, volumetric-modulated arc therapy in 45%, and tomotherapy in 25%. Image-guided radiotherapy (IGRT) for prostate was used in 71% of patients.

**Fig 1:**
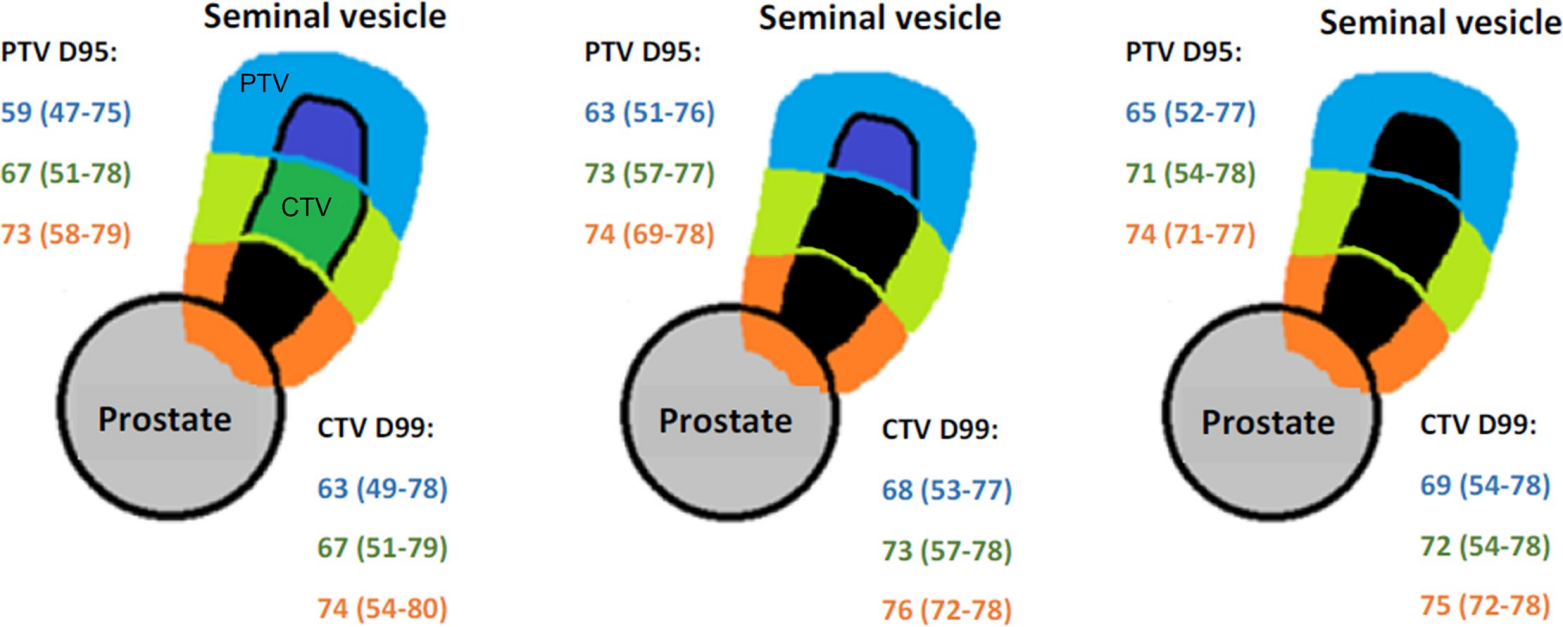
Dosimetric characteristics of each third of the seminal vesicle, depending on the length of involved seminal vesicles on MRI. Mean value (range) in Gy; Dx: dose delivered to x% of volume; PTV: planning target volume; CTV: clinical target volume; SV: seminal vesicle; SV PTV = SV CTV + 5 mm in all directions. Each SV was divided in three equal thirds, according to the cranio-spinal axis. The mean dose for each third of both SV is represented. The proximal third of the seminal vesicle is in orange, the second third is in green, and the distal third is in blue. The involved part of the seminal vesicle is in black.

The dosimetric characteristics of the prostate, the ISV, and uninvolved SV by each third of the SV appear in Fig 1 and Table 2. The dosimetric characteristics for the rectum and the bladder appear in Table 3. The rectum V72 was correlated with the prostate PTV D95 (r = 0.53, p < 0.01). The percentage of cases not respecting the dose constraints for the rectum and the bladder also appear in Table 3, ranging from 0% to 12% of patients, depending on the OAR and the recommendation.

**Table 2:** Doses to the prostate and the seminal vesicles, depending on the length of seminal vesicle involvement on MRI.

**PROSTATE**		
PTV D95	74 (70–76)	
CTV D99	75 (70–80)	
**INVOLVED SEMINAL VESICLES**		
**Part(s) of ISV**	**PTV D95**	**CTV D99**
If involvement limited at proximal 1/3	73 (58–79)	74 (54–80)
If involvement limited at proximal 2/3	74 (63–77)	75 (65–78)
If involvement in the entire SV	67 (54–72)	69 (55–78)
Whatever the involved part of ISV	72 (53–79)	73 (54–80)
**NOT INVOLVED SEMINAL VESICLES**		
**Part(s) of non-involved SV**	**PTV D95**	**CTV D99**
If non-involvement of the distal 2/3	63 (49–77)	65 (50–78)
If non-involvement of the distal 1/3	63 (51–76)	68 (53–77)
Whatever the non-involved part of SV	63 (49–77)	66 (50–78)

Mean value (range) in Gy. Dx: dose delivered to x% of the target volume; PTV: planning target volume; CTV: clinical target volume; ISV: involved seminal vesicles on MRI.

**Table 3:** Dosimetric characteristics of the rectum and the bladder according to the recommendations of the cooperative groups.

Vx	MEAN VALUES AND % OF PATIENTS OVER THE THREHOLD Vx VALUE	RECTUM	BLADDER
V50	Mean value (range) (Gy)	37 (10–94)	38 (6–96)
	% of patients with V50 ≥ 50% &	10	-
V60	Mean value (range) (Gy)	23 (5–61)	26 (2–71)
	% of patients with V60 ≥ 50%°#	0	7
	% of patients with V60 ≥ 35% &	6	-
V65	Mean value (range) (Gy)	18 (3–47)	21 (2–60)
	% of patients with V65 ≥ 50% #&	-	5
	% of patients with V65 ≥ 35% #	1	-
	% of patients with V65 ≥ 25% &	12	-
V70	Mean value (range) (Gy)	12 (1–27)	16 (0–51)
	% of patients with V70 ≥ 50%°	-	0
	% of patients with V70 ≥ 35% #&	-	6
	% of patients with V70 ≥ 20% #&	8	-
V72	Mean value (range) (Gy)	9 (0–22)	-
	% of patients with V72 ≥ 25%°	0	-
V75	Mean value (range) (Gy)	3 (0–14)	9 (0–40)
	% of patients with V75 ≥ 25% #&	-	5
	% of patients with V75 ≥ 15% #&	0	-
	% of patients with V75 ≥ 5% [23]	21	-

Vx: Dose–volume threshold recommendations (volume of organ receiving at least x Gy in %) according to the GETUG (°) (21); RTOG (#) [22]; QUANTEC (&) [20] or Fiorino et *al*.[23].

The mean duration of ADT was 36 months.

### Carcinological results and prognostic analysis

The median and mean follow-up were 26 and 36 months (95% CI: 33–39 months), respectively.

**Fig 2:**
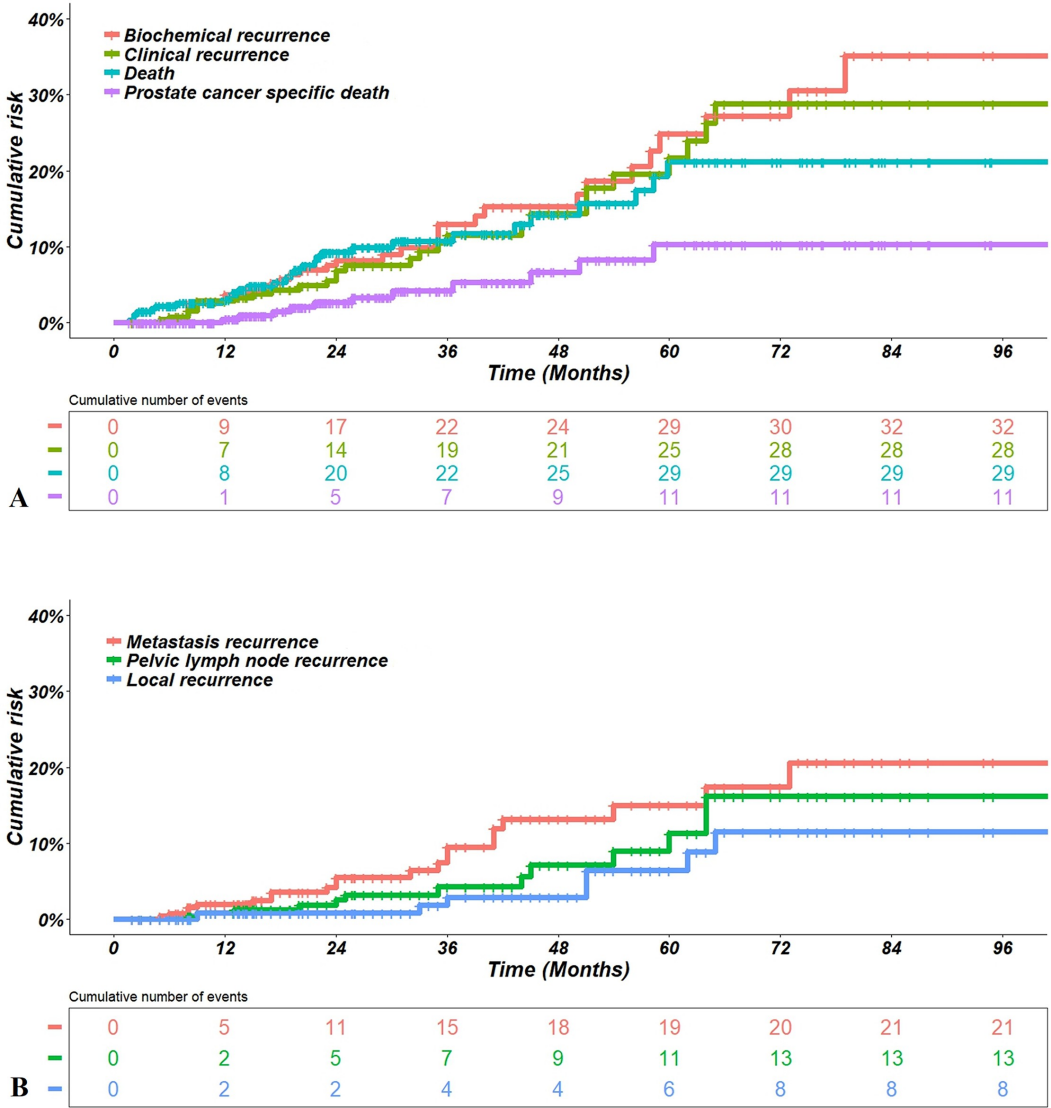
Carcinological results for T3b prostate cancer patients. (A)Results for biochemical and clinical recurrences and deaths. (B) Results for clinical recurrence detailed by local recurrence as the first event (local, pelvic lymph node, and metastasis). Clinical recurrence was defined as at least local or pelvic lymph node or metastasis recurrence.

Five-year biochemical and clinical recurrence rates were 24.8% (95% CI: 15.0–34.6%) and 21.7% (95% CI: 12.5–30.9%), respectively (Fig 2A). Regarding clinical recurrence, 5-year local, pelvic lymph node, and metastatic recurrence rates were 6.4% (95% CI: 0.6–12.2%), 11.3% (95% CI: 3.7–18.9%), and 15% (95% CI: 7.8–22.2%), respectively (Fig 2B). Five-year DFS, cause-specific survival, and overall survival rates were 65.8% (95% CI: 55.6–76.0%), 89.7% (95% CI: 82.9–96.5%) and 78.8% (95% CI: 70.2–87.4%), respectively (Fig 2A).

**Fig 3:**
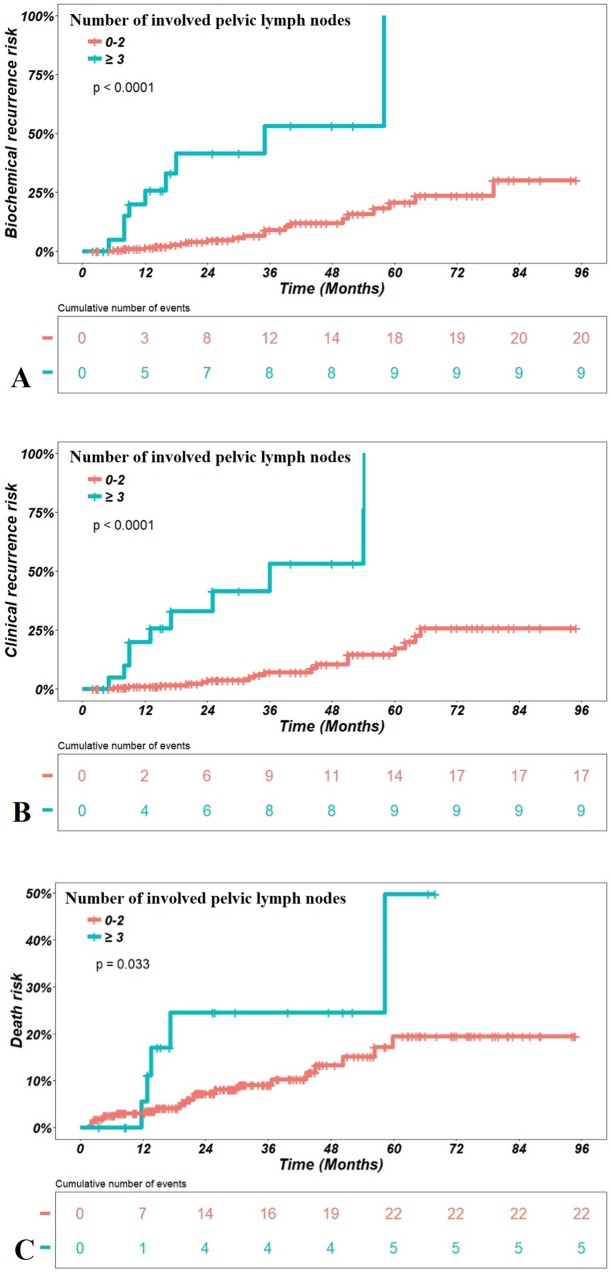
Impact of lymph node involvement (≤ 2 or ≥ 3 lymph nodes on MRI) on carcinological outcomes. (A)on biochemical recurrence, (B) on clinical recurrence, (C) on death. Clinical recurrence was defined as at least local or pelvic lymph node or metastasis recurrence. The p value has been calculated from the logrank test.

Considering the whole series, Table 4 presents the results of univariate analysis testing the impact of all parameters on each carcinological endpoint. Lymph node involvement on MRI increased significantly all the risks of recurrences (biochemical, clinical, local, lymph node and metastases) and deaths (Pca and overall) in univariate analysis (Table 4). Extracapsular extension, high number of prostate positive biopsies or ISV segments at MRI and high Gleason sore increased significantly the risk of overall or PCa specific death. Table 5 details the results of multivariate analysis. The number of involved pelvic lymph nodes ≥ 3 on MRI remained the only significant prognostic factor increasing the risk of occurrence of all carcinological endpoints, except overall death, with hazard ratios (HR) ranging from 5.3 to 12.9, and C-index ranging from 0.60 to 0.70. Fig 3 illustrates the impact of lymph node involvement (≤ 2 or ≥ 3 lymph nodes on MRI) on the endpoints. For overall death, the number of prostate positive biopsies remained the only significant factor (C-index = 0.71).

**Table 4:** Univariate analysis testing the impact of all parameters on all carcinological endpoints in the whole series.

Parameters		Biochemical recurrence		Clinical recurrence*		Local recurrence		Pelvic lymph node recurrence		Metastasis recurrence		Overall death		PCa specific death	
		p	HR (95% CI)	p	HR (95% CI)	p	HR (95% CI)	p	HR (95% CI)	p	HR (95% CI)	p	HR (95% CI)	p	HR (95% CI)
Center parameter		0.24		0.60		0.77		0.49		0.85		0.86		0.90	
Clinical parameter	Age	0.11		0.054		0.79		0.67		0.10		0.07		0.74	
Tumor parameters	T stage at DRE	0.90		0.64		0.63		0.96		0.63		0.62		0.41	
	PSA	0.78		0.98		0. 38		0.22		0.95		0.51		0.49	
	Number of D’Amico risk factors	0.59		0.40		0.48		0.43		0.81		0.51		0.09	
MRI parameters	Extracapsular extension	0.08		0.07		0.93		0.44		0.10		0.034	4.74 (1.13–20.00)	0.23	
	Bilateral prostate involvement	0.94		0.99		0.76		0.33		0.38		0.32		0.49	
	Bilateral SV involvement	0.08		0.08		0.39		0.41		0.13		0.08		0.07	
	Proximal ISV only (at least one SV)	0.72		0.93		0.14		0.17		0.73		0.66		0.21	
	Whole ISV (at least one SV)	0.73		0.97		0.62		0.86		0.75		0.19		0.13	
	Number of ISV segments #	0.84		0.49		0.73		0.98		0.42		0.031	1.30 (1.02–1.64)	0.06	
	Pelvic lymph node involvement	0.002	2.94 (1.47–5.87)	0.005	2.90 (1.38–6.03)	0.85		0.39		0.001	4.70 (1.95–11.36)	0.39		0.017	4.45 (1.30–15.23)
	Number of involved lymph nodes	<0.001	1.44 (1.24–1.67)	<0.001	1.47 (1.26–1.71)	0.43		0.014	1.38 (1.01–1.80)	<0.001	1.50 (1.30–1.76)	0.19		0.001	1.45 (1.16–1.81)
	Number of involved lymph nodes (≥3)	<0.001	8.20 (3.68–18.26)	<0.001	9.86 (4.28–22.69)	0.04	5.41 (1.06–27.47)	0.012	5.3 (1.43–19.6)	<0.001	12.95 (5.21–32.17)	0.038	2.78 (1.06–7.29)	0.001	7.60 (2.22–26.03)
Pathological parameters	Gleason score	0.28		0.12		0.31		0.27		0.19		0.14		0.020	2.33 (1.15–4.74)
	Number of positive biopsies in the prostate	0.60		0.32		0.89		0.68		0.18		0.003	1.18 (1.06–1.31)	0.08	
	Positive biopsies in SV°	0.26		0.28		0.58		0.49		0.37		0.92		0.53	
Dosimetric parameters	Dose prescribed to prostate	0.43		0.50		0.83		0.99		0.37		0.92		0.40	
	Dose prescribed to ISV	0.89		0.70		0.81		0.35		0.54		0.50		0.18	
	Dose prescribed to non-involved SV	0.74		0.94		0.18		0.30		0.23		0.18		0.33	
	Dose prescribed to non-involved pelvic lymph node	0.65		0.75		0.18		0.22		0.23		0.17		0.19	
	Dose prescribed to involved pelvic lymph node	0.64		0.60		0.95		0.48		0.64		0.66		0.34	
Systemic treatment parameter	ADT duration	0.18		0.10		0.86		0.003	1.04 (1.01–1.06)	0.40		0.10		0.21	

PCa: prostate cancer; DRE: digital rectal examination; SV: seminal vesicle; ISV: involved SV; ADT: androgen deprivation therapy. Each SV was divided in three equal segment in the cranio-spinal axis.

*Clinical recurrence was defined as at least local or pelvic lymph node or metastasis recurrence.

# The number of ISV could range from 1 (one proximal third of one ISV) to 6 (two whole ISV).

°Only 8% of patients had biopsy of the SV. The Cox model has been used. For significant p-values (p ≤0.05), hazard ratios (HR) with 95% confidence intervals (CI) are given.

**Table 5:** Multivariate analysis testing the impact of all parameters on all carcinological endpoints in the whole series and for patients without lymph node involvement on MRI.

Endpoints	Whole series				Patients without pelvic lymph node involvement			
	Parameters	p value	HR (95% CI)	C-index	Parameters	p value	HR (95% CI)	C-index
**Biochemical recurrence**	Number of involved lymph nodes (≥3)	<0.001	8.20 (3.68–18.26)	0.66	NI	-	-	-
**Clinical recurrence**	Number of involved lymph nodes (≥3)	<0.001	9.86 (4.28–22.69)	0.68	NI	-	-	-
**Local recurrence**	Number of involved lymph nodes (≥3)	0.04	5.41 (1.06–27.47)	0.66	NI	-	-	-
**Pelvic lymph node recurrence**	Number of involved lymph nodes (≥3)	0.012	5.30 (1.43–19.6)	0.60	NI	-	-	-
**Metastasis recurrence**	Number of involved lymph nodes (≥3)	<0.001	12.95 (5.21–32.17)	0.70	NI	-	-	-
**Overall death**	Number of positive biopsies in the prostate	0.003	1.18 (1.06–1.31)	0.71	Number of positive biopsies in the prostate	0.018	1.18 (1.03–1.35)	0.77
**PCa specific death**	Number of involved lymph nodes (≥3)	0.001	7.60 (2.22–26.03)	0.69	Gleason score	0.056*	3.34 (0.98–11.51)	0.81

PCa: prostate cancer; NI: not identified

*Trend towards statistical significance. Clinical recurrence was defined as at least local or pelvic lymph node or metastasis recurrence. The number of involved lymph nodes (≥3) was defined on MRI. The predictive performance of the Cox model was estimated by the C-index. Hazard ratios (HR) with 95% confidence intervals (CI) are given.

Considering only the patients without lymph node involvement on MRI, the high number of D’Amico risk factors or ISV segments on MRI or pathological positive prostate biopsies and extracapsular extension on MRI increased significantly the risk of death in univariate analysis (Table 6). In multivariate analysis, only the high number of prostate positive biopsies increased significantly the risk of death (HR = 1.18; C-index = 0.77) (Table 5). A high Gleason score increased at the limit of significance the risk of PCa specific death (HR = 3.34, p = 0.056; C-index = 0.81).

**Table 6:** Univariate analysis testing the impact of all parameters on carcinological endpoints for patients without lymph node involvement on MRI.

Parameters		Biochemical recurrence		Clinical recurrence*		Local recurrence		Pelvic lymph node recurrence		Metastasis recurrence		Overall death		PCa specific death	
		p	HR (95% CI)	p	HR (95% CI)	p	HR (95% CI)	p	HR (95% CI)	p	HR (95% CI)	p	HR (95% CI)	p	HR (95% CI)
Center parameter		0.11		0.30		0.92		0.94		0.23		0.88		0.97	
Clinical parameter	Age	0.37		0.28		0.65		0.99		0.62		0.48		0.37	
Tumor parameters	T stage at DRE	0.43		0.93		0.43		0.48		0.22		0.91		0.38	
	PSA	0.34		0.41		0.64		0.39		0.43		0.78		0.99	
	Number of D’Amico risk factors	0.98		0.44		0.37		0.71		0.64		0.046	1.90 (1.01–3.57)	0.28	
MRI parameters	Extracapsular extension	0.64		0.48		0.93		0.84		0.83		0.049	7.58 (1.01–56.80)	0.42	
	Bilateral prostate involvement	0.60		0.51		0.77		0.82		0.58		0.33		0.42	
	Bilateral SV involvement	0.51		0.55		0.95		0.51		0.96		0.15		0.17	
	Proximal ISV only (at least one SV)	0.61		0.97		0.25		0.39		0.41		0.78		0.91	
	Whole ISV (at least one SV)	0.58		0.81		0.67		0.58		0.81		0.27		0.31	
	Number of segment ISV #	0.89		0.77		0.82		0.21		0.84		0.014	1.46 (1.08–2.00)	0.33	
Pathological parameters	Gleason score	0.79		0.30		0.34		0.90		0.62		0.07		0.056	
	Number of positive biopsies in the prostate	0.54		0.69		0.89		0.28		0.25		0.018	1.18 (1.03–1.35)	0.97	
	Positive biopsies in the SV°	0.36		0.39		0.55		0.53		0.54		0.85		0.66	
Dosimetric parameters	Dose prescribed to prostate	0.24		0.21		0.80		0.56		0.22		0.21		0.45	
	Dose prescribed to ISV	0.44		0.54		0.85		0.17		0.79		0.88		0.53	
	Dose prescribed to non-involved SV	0.12		0.12		0.25		0.07		0.99		0.12		0.057	
	Dose prescribed to pelvic lymph node	0.73		0.71		0.59		0.43		0.95		0.24		0.18	
Systemic treatment parameter	ADT duration	0.13		0.06		0.71		0.07		0.15		0.13		0.43	

PCa: prostate cancer; DRE: digital rectal examination; SV: seminal vesicle; ISV: involved SV; ADT: androgen deprivation therapy. Each SV was divided in three equal segment in the cranio-spinal axis.

*Clinical recurrence was defined as at least local or pelvic lymph node or metastasis recurrence.

# The number of ISV could range from 1 (one proximal third of one ISV) to 6 (two whole ISV).

°Only 8% of patients had biopsy of the SV. The Cox model has been used. For significant p-values (p ≤0.05), hazard ratios (HR) with 95% confidence intervals (CI) are given.

### Urinary and digestive toxicity rates and predictors of toxicity

Acute urinary toxicity rates were 66% for grades 0–1, 31.4% for grade 2, and 2.6% for grade 3. One patient stopped the radiotherapy course because of grade 3 urinary toxicity. Five-year late urinary grade ≥ 2 toxicity rate was 31.5% (95% CI: 23.3–39.7). Five patients (1.8%) presented late urinary grade 3 toxicity, and none had grade 4 toxicity.

Considering the whole series, Table 7 details the results of univariate analysis testing the impact of all parameters on urinary grade ≥ 2 toxicity. The only significant predictive factors on acute and late toxicity were history of cardiovascular disease (HR = 2.45; AUC = 0.57) and ADT duration (HR = 0.98; AUC = 0.55), respectively. Considering only the patients without lymph node involvement on MRI, the same significant predictive factors were found (Table 8).

**Table 7:** Univariate analysis testing the impact of all parameters on grade ≥ 2 toxicity in the whole series.

Parameters		Acute grade ≥ 2 toxicity				Late grade ≥ 2 toxicity			
		Urinary		Digestive		Urinary		Digestive	
		p	HR (95% CI)	p	HR (95% CI)	p	HR (95% CI)	p	HR (95% CI)
Center parameter		0.027	NA	0.07		0.08		0.38	
Clinical parameter	Age	0.73		0.85		0.47		0.016	1.08 (1.02–1.16)
	Arterial hypertension	0.65		0.30		0.68		0.018	2.87 (1.20–6.84)
	Cardiovascular disease	0.018	2.45 (1.16–5.15)	0.23		0.79		0.83	
	Diabetes	0.50		0.84		0.78		0.52	
	Anticoagulant treatment	0.30		0.58		0.44		0.71	
	History of pelvic surgery	0.57		0.74		0.87		0.20	
Tumor parameters	T stage at DRE	0.91		0.54		0.61		0.28	
MRI parameters	Bilateral SV involvement	0.66		0.73		0.68		0.42	
	Proximal ISV only (at least one SV)	0.24		0.39		0.71		0.047	0.42 (0.17–0.98)
	Whole ISV (at least one SV)	0.99		0.22		0.79		0.51	
	Number of segment ISV #	0.70		0.60		0.99		0.11	
	Pelvic lymph node involvement	0.35		0.41		0.059		0.97	
Dosimetric target volumes parameters	Dose prescribed to prostate	0.45		0.24		0.85		0.47	
	Dose prescribed to ISV	0.92		0.12		0.74		0.36	
	Dose prescribed to non-involved SV	0.18		<0.001	1.09 (1.04–1.13)	0.19		0.66	
	Dose prescribed to non-involved pelvic lymph node	0.79		0.12		0.69		0.005	1.11 (1.03–1.19)
	Dose prescribed to involved pelvic lymph node	0.17		0.41		0.16		0.52	
Dosimetric organ at risks parameters	Rectum V40	NA		0.85		NA		0.45	
	Rectum V50	NA		0.88		NA		0.93	
	Rectum V60	NA		0.70		NA		0.96	
	Rectum V65	NA		0.87		NA		0.99	
	Rectum V70	NA		0.85		NA		0.97	
	Rectum V75	NA		0.20		NA		0.14	
	Bladder V50	0.80		NA		0.22		NA	
	Bladder V60	0.98		NA		0.27		NA	
	Bladder V70	0.82		NA		0.40		NA	
Systemic treatment parameter	ADT duration	0.12		0.68		0.016	0.98 (0.96–0.99)	0.57	

DRE: digital rectal examination; SV: seminal vesicle; ISV: involved SV; Vx: volume of organ receiving at least x Gy in %; ADT: androgen deprivation therapy; NA: not applicable. Each SV has been divided in three segments in the cranio-spinal axis.

# The number of ISV could range from 1 (one proximal third of on ISV only) to 6 (two whole ISV). The logistic regression test has been used for acute toxicity. The Cox model has been used for late toxicity. For significant p-values (p ≤0.05), hazard ratios (HR) with 95% confidence intervals (CI) are given.

**Table 8:** Univariate analysis testing the impact of all parameters on grade ≥ 2 toxicity for patients without lymph node involvement on MRI.

Parameters		Acute grade ≥ 2 toxicity				Late grade ≥ 2 toxicity			
		Urinary		Digestive		Urinary		Digestive	
		p	HR (95% CI)	p	HR (95% CI)	p	HR (95% CI)	p	HR (95% CI)
Center parameter		0.21		0.59		0.26		0.73	
Clinical parameter	Age	0.92		0.54		0.54		0.004	1.13 (1.04–1.23)
	Arterial hypertension	0.69		0.13		0.92		0.007	4.84 (1.55–15.11)
	Cardiovascular disease	0.035	2.33 (1.06–5.13)	0.34		0.96		0.92	
	Diabetes	0.69		0.34		0.92		0.68	
	Anticoagulant treatment	0.33		0.33		0.42		0.37	
	History of pelvic surgery	0.68		0.66		0.81		0.68	
Tumor parameters	T stage at DRE	0.42		0.47		0.89		0.37	
MRI parameters	Bilateral SV involvement	0.90		0.30		0.67		0.59	
	Proximal ISV only (at least one SV)	0.20		0.83		0.63		0.22	
	Whole ISV (at least one SV)	0.69		0.52		0.29		0.29	
	Number of segment ISV #	0.61		0.49		0.31		0.92	
Dosimetric target volumes parameters	Dose prescribed to prostate	0.49		0.07		0.50		0.43	
	Dose prescribed to ISV	0.97		0.25		0.75		0.86	
	Dose prescribed to non-involved SV	0.12		0.018	1.06 (1.01–1.12)	0.52		0.32	
	Dose prescribed to pelvic lymph node	0.95		0.15		0.76		0.007	1.25 (1.06–1.47)
Dosimetric organ at risks parameters	Rectum V40	NA		0.57		NA		0.86	
	Rectum V50	NA		0.76		NA		0.74	
	Rectum V60	NA		0.94		NA		0.87	
	Rectum V65	NA		0.96		NA		0.82	
	Rectum V70	NA		0.87		NA		0.84	
	Rectum V75	NA		0.23		NA		0.47	
	Bladder V50	0.76		NA		0.40		NA	
	Bladder V60	0.95		NA		0.09		NA	
	Bladder V70	0.97		NA		0.11		NA	
Systemic treatment parameter	ADT duration	0.21		0.73		0.013	0.97 (0.96–0.99)	0.56	

DRE: digital rectal examination; SV: seminal vesicle; ISV: involved SV; Vx: volume of organ receiving at least x Gy in %; ADT: androgen deprivation therapy; NA: not applicable. Each SV has been divided in three segments in the cranio-spinal axis.

# The number of ISV could range from 1 (one proximal third of on ISV only) to 6 (two whole ISV). The logistic regression test has been used for acute toxicity. The Cox model has been used for late toxicity. For significant p-values (p ≤0.05), hazard ratios (HR) with 95% confidence intervals (CI) are given.

Acute digestive toxicity rates were 86.8% for grades 0–1, 11% for grade 2, and 2.2% for grade 3. Five-year late digestive grade ≥ 2 toxicity rate was 12% (95% CI: 6.8–17.2%). Five patients (1.8%) presented late digestive grade 3 toxicity and none had grade 4 toxicity.

**Fig 4:**
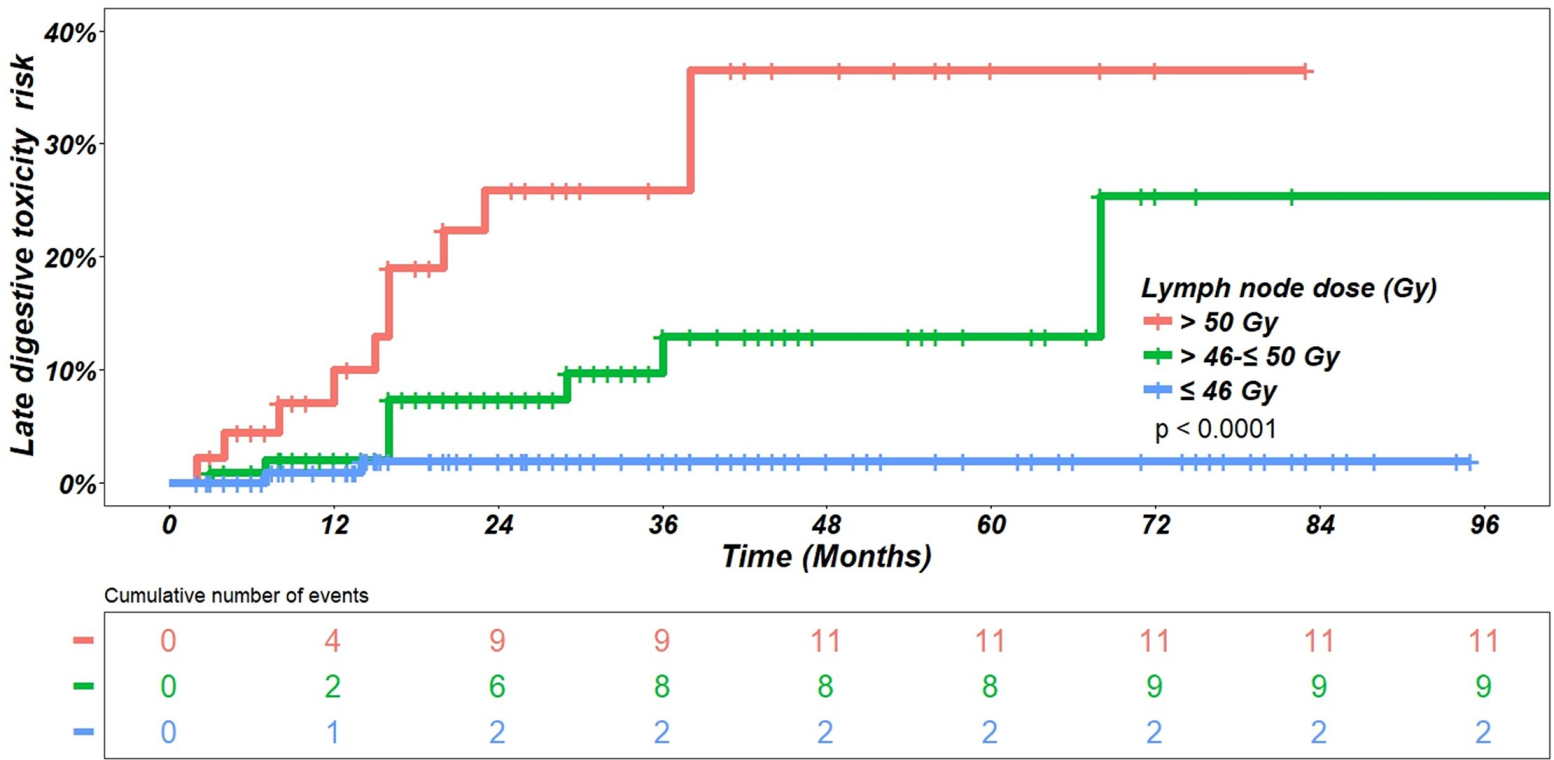
Impact of pelvic node dose on late digestive toxicity (grade ≥ 2). The p value has been calculated from the logrank test.

Considering the whole series, Table 7 details univariate analysis testing the impact of all parameters on digestive grade ≥ 2 toxicity. The only significant predictive factor on acute toxicity was the non-involved SV dose (HR = 1.09; AUC = 0.73). The significant predictive factors on late digestive toxicity were age (HR = 1.08), arterial hypertension (HR = 2.87), proximal ISV only on MRI (HR = 0.42) and non-involved pelvic lymph node dose (HR = 1.11). Table 9 details the results of multivariate analysis. The retained significant predictive factors on late digestive toxicity were age (HR 1.09) and pelvic lymph node dose (HR = 1.12) with an AUC of 0.78. Fig 4 illustrates the impact of pelvic node dose on late digestive toxicity (grade ≥ 2). Considering only the patients without lymph node involvement on MRI, univariate analysis retained the same significant predictive factors except the proximal ISV only (Table 8). In multivariate analysis, the significant predictive factors were age, arterial hypertension and pelvic lymph node dose with an AUC of 0.84 (Table 9).

**Table 9:** Multivariate analysis testing the impact of all parameters on grade ≥ 2 toxicity in the whole series and for patients without lymph node involvement on MRI.

Endpoints		Whole series				Patients without lymph node involvement			
		Parameters	p value	HR (95% CI)	AUC/C-index	Parameters	p value	HR (95% CI)	AUC/C-index
**Acute toxicity**	**Urinary toxicity grade ≥ 2**	Cardiovascular disease	0.018	2.45 (1.16–5.15)	0.57	Cardiovascular disease	0.035	2.33 (1.06–5.13)	0.58
	**Digestive toxicity grade ≥ 2**	Dose prescribed to non-involved SV	<0.001	1.09 (1.04–1.13)	0.73	Dose prescribed to non-involved SV	0.018	1.06 (1.01–1.12)	0.69
**Late toxicity**	**Urinary toxicity grade ≥ 2**	ADT duration	0.016	0.98 (0.96–0.99)	0.55	ADT duration	0.013	0.97 (0.96–0.99)	0.56
	**Digestive toxicity grade ≥ 2**	Age	0.010	1.09 (1.02–1.16)	0.78	Age	0.020	1.10 (1.02–1.20)	0.84
		Dose prescribed to non-involved pelvic lymph node	0.004	1.12 (1.04–1.22)		Arterial hypertension	0.040	3.33 (1.06–10.50)	
						Dose prescribed to pelvic lymph node	0.026	1.21 (1.02–1.43)	

ADT: androgen deprivation therapy; AUC: Area under the ROC curve. A logistic regression test has been used for acute toxicity and a cox model for late toxicity. For significant p-values (p ≤0.05), hazard ratios (HR) with 95% confidence intervals (CI) are given. The predictive capabilities of the logistic regression and Cox models were estimated using the area under the receiver operating characteristic (ROC) curve (AUC) and the C-index, respectively.

## Discussion

This study is the only one reporting experience of IMRT for specifically T3b PCa. Escalating the dose in the ISV potentially exposes the patient to an increased risk of toxicity. In our study, IMRT allowed dose escalation in ISV while respecting the dose constraints for the OARs in more than 85% of cases. The dose to the ISV depended, however, on the involved third of the SV, the distal involved third of the SV receiving less dose than the proximal third of the SV close to the prostate (Fig 1). Moreover, the uninvolved SV also received a high dose (mean dose ≥ 63 Gy) (Table 2), depending also on the uninvolved SV third (Fig 1). By escalating the dose (≥ 76 Gy) in the prostate only with 3D-CRT, the literature shows that the 5-year urinary and digestive grade ≥ 2 toxicity rates range from 12% to 21% and from 7% to 21%, respectively [19,21,29]. With IMRT, the 5-year digestive toxicity rate are reduced to less than 10% [30]. By escalating the dose in both the prostate and the whole or a part of the SV, the toxicity appears increased, with 5-year urinary and digestive grade ≥ 2 toxicity rates ranging with 3D-CRT from 12% to 39% and from 26% to 32%, respectively [31,32], and with IMRT from 12% to 16% and from 4% to 21%, respectively [22,33]. Regarding digestive toxicity, these results by appear, therefore, comparable to our study (5-year rate of 12%). Our rate of urinary toxicity appears, however, quite higher (5-year rate of 31.5%), despite the systematic use of IMRT and the use of IGRT in the majority of cases. Reducing SV PTV below 5 mm to decrease toxicity appears to be a poor option [34]. Indeed, the SV present deformations and move independently from the prostate, with a motion magnitude larger than the prostate, even if the tumor infiltration within the SV reduces their mobility [35,36].

Table 10 summarizes the literature, reporting results of treatment for specifically T3b PCa. The large majority of treatments are based on surgical experience in selected T3b PCa patients. Our treatment results appear thus comparable to surgical series in which the 5-year biochemical DFS rate ranges from 21% to 70% (66% in our study), and the 5-year cause-specific survival rate ranges from 94% to 98% (90% in our study) [8–13,37–41]. Such retrospective comparison between treatments must be considered with caution due in particular to biases in patient and tumor selections (highest age, PSA, and Gleason score in our RT series compared to the surgical studies).

**Table 10:** Review of the literature reporting treatment for T3b prostate cancer.

TREATMENT	STUDIES	Nb	PATIENTS AND TUMOR CHARCTERISTICS				ADJUVANT TREATMENT			CARCINOLOGICAL RESULTS				
			Age (Y)	PSA (ng/mL)	Gleason score (%)	N1 (%)	ADT (%)	RT (%)	ADT and RT (%)	Follow-up (months)	DFS (%)	MFS (%)	CSS (%)	OS (%)
Surgery	Moschini [37]	3279	65	22	6: 17% 7: 46% 8–10: 37%	38	36	14	8	148	5-Y: 50 10-Y: 36	-	5-Y: 94 10-Y: 86	5-Y: 90 10-Y: 73
	Hubanks [8]	1132	66	10	6: 27% 7: 49% 8–10: 24%	No	30	12	-	127	10-Y: 41	10-Y: 81	5-Y: 96 10-Y: 89	10-Y: 59
	Pierorazio [9]	989	60	10	6 : 10% 7 : 55% 8–10 : 35%	25	28	5	-	160	5-Y: 38 10-Y : 25	5-Y: 83 10-Y: 70	5-Y: 94 10-Y: 81	-
	Secin [10]	387	62	11	6–7: 83% 8–10: 17%	24	No	No	No	68	5-Y: 38	-	5-Y: 96 10-Y: 85	-
	Siddiqui [11]	382	66	4	6: 27% 7: 48% 8–10: 25%	No	50	No	No	120	5-Y: 70 with ADT and 32 without 10-Y: 60 with and 16 without	5-Y: 93 with ADT and 88 without 10-Y: 88 with ADT and 78 without	5-Y: 98 with ADT and 97 without 10-year: 94 with ADT and 87 without	10-Y: 75% with ADT and 69% without
	Jang [12]	350	67	16	6–7: 37% 8–10: 63%	20	No	No	No	69	5-Y: 21	-	-	-
	Bastide [13]	199	64	13	6: 11% 7: 75% 8–10: 25%	No	38,	46	25	60	5-Y: 48	-	-	5-Y: 93
	Pagano [38]	180	64	9	6–7: 50% 8: 20% 9–10: 30%	12	No	No	No	27	5-Y: 60	-	-	-
	SWOG 8794 [39]	139	65	-	-	No	No	51	No	146	5-Y: 49 10-Y: 22	5-Y: 74	-	5-Y: 86 10-Y: 61
	Salomon [40]	137	64	17	6–7: 63% 8–10: 37%	No	No	No	No	59	5-Y : 34 10-Y : 10	-	-	-
	Freedland [41]	135	63	18	6: 25% 7: 45% 8–10: 30%	No	No	No	No	44	5-Y : 36	-	-	-
EBRT + BT	Koutrouvelis* [51]	37	68	20	6: 19% 7 : 56% 8 : 25%	No	No	-	-	24	2-Y: 79	-	-	-
	Stone [47]	52	70	-	6: 25% 7 : 44% 8–10 : 31%	No	All	-	-	56	5-Y: 70 10-Y: 64	-	10-Y: 91	10-Y: 83
	Rades [48]	38	-	-	6–7: 73% 8–10 : 27%	No	All, 5 months	-	-	37	3-Y: 79 and 57 with and without dose escalation in SV	-	-	-
	Tsamura [49]	80	72	47	6: 5% 7: 42% 8: 24% 9–10: 29%	No	All, 36 months	-	-	74	5-Y: 82 10-Y: 64	-	5-Y: 95 10-Y: 91	5-Y: 88 10-Y: 84
	Stone [50]	53	66	-	6: 19% 7 : 36% 8–10 : 45%	No	All	-	-	109	10-Y: 61	10-Y: 78	10-Y: 77	-
EBRT only	Our series	276	69	27	6: 7% 7: 51% 8–10: 42%	26%	All	-	-	36	5-Y: 66	-	5-Y: 90	5-Y: 79

Selected surgery studies comprise a minimum of 100 patients, with Gleason score and pelvic lymph node involvement analyzed on pathological examination. Mean values are given. Nb: number of patients; Y: year; N1: pelvic lymph node involvement on pathological examination in case of surgery or on imaging (CT or MRI) in case of radiotherapy; ADT: androgen deprivation therapy; EBRT: external beam radiotherapy; BT: brachytherapy; DFS: disease-free survival; MFS: metastasis-free survival; CSS: cause-specific survival; OS: overall survival.

*BT only.

The best mean for dose escalation is BT. Indeed, the superiority of the combined treatment over EBRT has been demonstrated only in series comprising both intermediate and high-risk PCa in terms of biochemical control in three randomized studies [42–44], of metastases, and even of overall survival in large national databases [45]. More recently, in high-risk PCa and after adjustment for PCa prognostic factors, other medical conditions, and socioeconomic factors, the combination of BT to EBRT or radical prostatectomy have been shown to provide higher overall survival than EBRT combined with ADT [46]. The proportion of T3 cancer treated by surgery was, however, small in this study. Only five studies, comprising a relatively small number of patients (≤ 80), reported results of the combination of EBRT and brachytherapy (BT) [47–50] or the use of BT only [51] for T3b PCa. Despite technique considerations with difficulties to ensure needles to the distal parts of SV, more than 80% of the entire SV volume received a dose escalation [48]. Furthermore, BT with dose escalation in SV compared to BT without dose escalation in SV increased to 22% of the 3 year DFS [48]. Our treatment results appear, therefore, slightly lower than those reported for the combined treatment, with 5-year DFS and cause-specific survival rates ranging from 70% to 82% and around 95% versus 66% and 90% in our study, respectively. However, these BT series excluded patients with lymph node involvement at imaging.

The incidence of lymph node involvement in T3b PCa is particularly high, ranging from 12% to 38% in surgical series [8–13,37–41] and 26% in our series (on MRI). In surgical studies, the lymph node involvement is the most significant prognostic factor of biochemical recurrence (HR = 2.1), DFS (HR = 2.9), and cause-specific survival (HR = 2.4) [9,14]. The lymph node involvement was also identified in our study as the only significant prognostic factor in multivariate analysis on biochemical recurrence (HR = 2.9), clinical recurrence (HR = 2.9), and PCa death (HR = 4.4). Moreover, a lymph node number greater than or equal to three increased dramatically the risk of clinical recurrence (HR = 9.9) and death (HR = 2.8), suggesting, therefore, to add another systemic treatment to this subgroup of patients such as chemotherapy or second-generation hormone therapy. This threshold in the number of positive lymph nodes has also recently been reported to affect patient outcome after PCa radiotherapy [52]. Moreover, the benefit of radiotherapy can also be discussed for patients with positive lymph nodes. Indeed, even if there is a lack of randomized evidence, retrospective studies suggest that radiotherapy may improve survival for the subgroup of patients [53–57]. Furthermore, among men with pN1 PCa (at radical prostatectomy), the subgroup benefiting from radiotherapy is the one with one to two positive nodes, pathological Gleason score 7–10, and pT3b/4 disease or positive surgical margins [58].

Other poor prognosis factors, such as a high Gleason score (≥ 8), high tumor infiltration on prostate biopsy and extracapsular extension were also identified as poor prognostic factors in several surgical and BT series [8,10,14,49,59], as well as in our series in univariate analysis.

Our study contains several limitations. The follow-up is short (26 months), explained by the recent generalization and accessibility of MRI at initial staging. Urinary toxicity must be therefore interpreted with precaution, knowing the possibility of late events. Moreover the analysis is retrospective, justifying the analysis of high grades of toxicity only (grade ≥ 2). Finally, a very small proportion of patients (8%) had a SV biopsy, confirming the ISV. Our patients are likely, however, to have true pathological ISV, as suggested by the high specificity of MRI for ISV [3]. The presence of lymph nodes on MRI was, nevertheless, a poor prognostic factor.

In conclusion, IMRT combined with IGRT allows delivery of a high curative dose (≥ 70 Gy) to the ISV in the majority of cases, while rarely exceeding dose-constraint recommendations at the planning. Acute and late-grade ≥ 2 digestive toxicity appears moderate (rates < 15%), whereas urinary toxicity is higher (> 30%) and slightly increased, compared to the reported toxicity after high dose delivered to the prostate only. Local control is high (94%), and the main pattern of recurrence is metastasis, mostly related to the lymph node involvement on MRI at diagnosis.

## Supporting information

S1 Dataset Dataset for this study. (XLSX)
